# Clinical Features and Surgical Outcomes of Posterior Segment Intraocular Foreign Bodies in Children in East China

**DOI:** 10.1155/2018/5861043

**Published:** 2018-06-25

**Authors:** Ting Zhang, Hong Zhuang, Keyan Wang, Gezhi Xu

**Affiliations:** ^1^Department of Ophthalmology, Eye, Ear, Nose and Throat Hospital, Fudan University, Shanghai 200031, China; ^2^Shanghai Key Laboratory of Visual Impairment and Restoration, Eye, Ear, Nose and Throat Hospital, Fudan University, Shanghai 200031, China

## Abstract

**Purpose:**

To report the long-term follow-up results of posterior segment intraocular foreign body (IOFB) removal in children and to determine the prognostic factors for visual outcome.

**Methods:**

Design: retrospective, noncomparative, interventional case series; a single tertiary care center study. Participants or samples: eleven eyes (11 patients) under 16 years of age with posterior segment IOFB injuries from May 2014 to November 2017. Main outcome measures: clinical features of injury, visual acuity, and complications.

**Results:**

The mean age was 6.8 years, and the mean follow-up was 20.2 months. The main IOFB sources were accidental penetration of the eye by materials in the playground (6 cases) or by pencil lead at school (4 cases). The mean IOFB size was 3.8 (range 1–6) mm. At the last visit, the visual acuities were 20/40 or better in 40.0% of patients and better than 20/200 in 70.0%. Poor visual outcome was correlated with intraoperative rhegmatogenous retinal detachment (*P*=0.0083). Postoperative complications included elevated transient intraocular pressure, retinal redetachment, and secondary glaucoma.

**Conclusions:**

The clinical features of pediatric posterior segment IOFBs suggest insufficient awareness of such injuries both on the playground and at school. Visual outcomes from surgical treatment were relatively favorable in this series.

## 1. Introduction

Ocular trauma is a leading cause of noncongenital monocular blindness in children, and it imposes a significant economic burden on society [1]. In adults, intraocular foreign bodies (IOFBs) account for approximately one-third of open globe injuries and most commonly affect the posterior segment of the eye [2, 3]. They generally occur at workplaces. The most common IOFB is metallic pieces produced from hammering. Foreign body injuries are also common in children [4]; however, there are few reports of IOFBs in patients aged < 16 years. Here, we report the clinical features and surgical outcomes in consecutive pediatric patients with posterior segment IOFBs who visited the Eye, Ear, Nose and Throat Hospital of Fudan University, the largest tertiary referral eye center in East China that handles almost all IOFB cases in this region.

## 2. Methods

### 2.1. Data Collection

This was a retrospective study designed to assess the characteristics and surgical outcomes of posterior segment IOFB injuries in children and possible prognostic factors for visual outcome. The posterior segment is the back two-thirds of the eye that includes the vitreous, retina, choroid, and optic nerve. Inclusion criteria were consecutive patients aged less than 16 years who were admitted to the Eye, Ear, Nose and Throat Hospital between January 2014 and December 2017 for posterior segment IOFB injuries. Exclusion criteria were patients older than 16 years or patients with only anterior segment or intraorbital foreign bodies. Patients with suspected IOFB preoperatively were also excluded with no final evidence of IOFB after imaging test or surgical treatment. The study was conducted in accordance with the principles of the Declaration of Helsinki and approved by the Ethics Committee of the Eye, Ear, Nose and Throat Hospital. Informed consent was obtained from each patient's parents.

At presentation to the hospital, a detailed medical history was collected from each child and their guardians, and each child underwent a complete ophthalmologic examination including assessment of visual acuity (VA), pupillary reflex evaluation, slit-lamp examination, and fundus examination. The interval between IOFB injury and surgery, type of injury, number of IOFBs, IOFB type and size, entry site of the IOFB, concomitant ocular injuries, presence of endophthalmitis, IOFB extraction route, and details of vitrectomy were recorded. Computed tomography (CT) of the orbits without contrast was performed preoperatively to confirm and localize the IOFBs. Helical CT was performed through the orbits in three planes (axial, sagittal, and coronal) with slice thickness of 0.75 mm. If the primary wound entry had already been closed, B scan ultrasonography was also used to confirm and localize the IOFB and evaluate the vitreous and retina. Ultrasound biomicroscopy was used to evaluate the IOFBs if CT indicated the presence of an IOFB in the ciliary body. The International Society of Ocular Trauma Classification [5, 6] and Birmingham Eye Trauma Terminology [7] systems were applied in the present study. The Ocular Trauma Score (OTS) predictions were compared with the actual visual outcomes, as performed by Yu Wai Man and Steel [8].

Postoperative follow-up visits were scheduled at 1, 7, and 14 days and 1, 2, 3, 6, 12, 24, and 36 months after surgery for each patient and included VA testing, intraocular pressure measurement, and a comprehensive ophthalmic examination. VA values obtained from the Snellen eye test were converted into the logarithm of the mean angle of resolution (logMAR) units for analysis. The VAs for hand motion, light perception, and no light perception were assigned logMAR values of 2.6, 3, and 4, respectively. Follow-up was scheduled after any additional surgeries.

The diagnosis of endophthalmitis was based on the clinical findings of presence of corneal edema, anterior chamber cells, hypopyon, and inflammation in the vitreous. The diagnosis of panophthalmitis was based on the ophthalmic examination findings of presence of corneal edema, hypopyon, severe vitritis, and edema of the eye walls, as well as an obvious elevation in white blood cell counts. Topical levofloxacin 0.5% and tobramycin 0.3% eye drops were applied preoperatively every 30 minutes to eyes with suspected infection. Intravitreal injections of ceftazidime (2.25 mg/0.1 ml) and vancomycin (1 mg/0.1 ml) were administered to eyes with endophthalmitis after the initial surgery to remove the IOFB. Intravenous ceftazidime (50 mg/kg twice daily) was administered for 3 days postoperatively to patients with endophthalmitis or panophthalmitis. The white blood cell count of patients with panophthalmitis was measured every other day until it decreased to within the normal range. The antibiotic therapy was adjusted accordingly when microorganisms were detected in cultures of vitreous samples or extracted IOFBs. Patients with no signs of infection did not receive intravitreal or systemic antibiotics. Topical prednisolone acetate 0.1% and levofloxacin 0.5% eye drops three times daily for 4 weeks postoperatively were prescribed for all eyes.

### 2.2. Surgical Procedures

All patients with IOFBs received surgical treatment within 1 day after admission to our hospital. Conventional three-port 20-gauge pars plana vitrectomy (PPV) was performed. An undiluted vitreous sample from eyes with suspected endophthalmitis was obtained at initiation of PPV for Gram staining and culture. Media opacities including hyphema, hypopyon, or traumatic cataract were removed prior to performing core vitrectomy. If the IOFB was suspended in the vitreous gel, adhesions surrounding the IOFB were dissected around the perimeter, and the IOFB was then extracted via scleral or limbal incision. After removal of an intravitreal IOFB, artificial posterior vitreous detachment was performed. However, if there were significant signs of vitritis or retinitis, excision of the hyaloid face was performed with extreme caution or even deferred for subsequent surgery. If retinal impact sites were present, with or without the IOFBs embedded in the impact sites, vitreous-retina adhesion around these areas was eliminated thoroughly, and the area around the retinal impact sites was treated with laser photocoagulation before removal of the IOFB.

The extraction strategy for the IOFBs was determined preoperatively, based mainly on the size and composition of the IOFB. Ferromagnetic IOFBs were removed bimanually using an intraocular magnet and forceps. Nonferrous or organic IOFBs were removed using foreign body forceps, and heavier-than-water liquid was introduced if necessary to protect the macula. Where possible, smaller foreign bodies were removed after enlargement of the sclerotomy site. Large IOFBs were removed via a new limbal incision.

After extraction, the IOFB was sent for microbiological culture, the limbal incision or enlarged scleral incision was sutured, and a complete retinal examination using scleral depression was conducted. If rhegmatogenous retinal detachment (RRD) was present, PPV using perfluoropropane or silicone oil tamponade was performed. The decision to use gas or oil tamponade was made by the surgeon, based on issues including the severity of endophthalmitis and the number and location of retinal breaks. The postoperative face-down position could not be accomplished by the children and thus was not mandatory. Removal of the silicone oil was scheduled for 3 months after the primary vitrectomy. Aphakic eyes with a postoperative VA better than 20/200 received intraocular lens implantation at least 3 months after successful vitrectomy.

Evisceration was indicated in cases of panophthalmitis. The cornea was excised completely following peritomy, and the intraocular contents and IOFB were removed using an evisceration spoon. The IOFB was sent for microbiological culture to detect pathogens, and the contents of the eye were sent for histopathological examination. Any remaining pigment was removed by scrubbing with cotton-tipped applicators soaked in 95% alcohol. Irrigation was then performed to remove the residual pigment and alcohol. A scleral shell was envelope-sutured, Tenon's capsule was closed, and the conjunctiva was sutured with burying of the knots. A conformer was then placed inside the eyelid. A hydroxyapatite implant was planned at least 3 months after the infection had subsided.

### 2.3. Statistical Analysis

Statistical analysis was performed using SPSS for Macintosh (version 21, SPSS Inc., Chicago, IL, USA). Fisher's exact test was used for comparisons of categorical variables. The preoperative and postoperative best-corrected visual acuity (BCVA) values were compared using the nonparametric Wilcoxon matched-pairs signed-rank test. A two-tailed *P* value < 0.05 was considered to indicate statistical significance in all tests.

## 3. Results

Eleven eyes in seven boys (63.6%) and 4 girls (36.4%; mean age 6.8 ± 3.0 years; range 2–12) were included in the study (Table 1). The most common causes of IOFB were accidental penetration of the eye by various materials in the playground (6 patients) and by pencil lead at school (4 patients).

The median interval from injury to IOFB removal was 2 days (mean 6.7, range 0.5–50) This was irrespective of the presence of vitritis (2.1 ± 1.7 days in 5 eyes with endophthalmitis or panophthalmitis versus 10.6 ± 19.4 days in 6 eyes with no signs of infection; *P*=0.18).

All injuries were type C IOFB according to the International Society of Ocular Trauma classification system and the OTS score was evaluated for each patient (Table 2). The entry site was zone I in eight eyes (72.7%) and zone II in three eyes (27.3%). Ten eyes (90.9%) had traumatic cataracts, four eyes (36.4%) had retinal tears, four eyes (36.4%) had suspected endophthalmitis, two eyes (18.2%) had vitreous hemorrhage, and one eye had panophthalmitis (9.1%) at presentation.

The OTS was available in 9 children. As shown in Table 3, the OTS predicted visual survival (LP or better) in 6 out of 8 children and no vision (NPL or enucleation) in 1 out of 1 child. Its sensitivity to predict visual survival and specificity to predict no vision was 75.0% and 100%, respectively. Similarly, the OTS predicted minimal to severe visual loss (20/20 to 3/60) in 6 out of 7 children and profound visual loss (worse than 3/60) in 2 out of 2 children. Its sensitivity to predict minimal to severe visual loss and specificity to predict profound vision loss was 85.7% and 100%, respectively.

The IOFBs were located in the retina in three eyes (27.3%), ciliary body in two eyes (18.2%), and vitreous in six eyes (54.5%). The composition of the IOFB was iron in four eyes, graphite in four eyes, wood in one eye, stone in one eye, and plastic (two pieces) from a fireworks explosion in one eye. In the eye with panophthalmitis, the IOFB was a stone thrown up with mud by exploding fireworks.

The IOFBs were removed via the scleral incision in PPV in 8 (72.3%) of 11 eyes, via limbal incision after extraction to the anterior chamber in 2 eyes (18.2%), and by evisceration in 1 eye (9.1%). A single IOFB was present in 10 eyes (90.9%) and multiple IOFBs in 1 eye (9.1%). The size of the IOFB varied from 2 × 2 × 1 mm to 6 × 6 × 4 mm, and the mean length was 3.8 ± 1.5 mm.

As shown in Table 4, cataract extraction combined with PPV, anterior PPV, and evisceration of the eye were performed in eight eyes (72.3%), two eyes (18.2%), and one eye (9.1%), respectively. In all cases, the IOFBs were removed during the initial surgery. Seven eyes underwent additional surgeries to treat complications, remove silicone oil, or restore VA; of these seven eyes, four underwent secondary implantation of an intraocular lens, one underwent implantation of a hydroxyapatite prosthesis, one underwent four procedures for recurrent retinal detachment, and one underwent simple silicone oil removal. Two eyes were prescribed rigid gas-permeable contact lenses after removal of the corneal sutures. Seven eyes with a postoperative VA better than 20/200 were treated for amblyopia.

The mean follow-up period was 20.2 (range 3–42) months. Preoperative and final VA data were available for 9 and 10 eyes, respectively (Tables 1 and 4). The mean preoperative and final VAs were 1.37 ± 1.35 (range 0–4) logMAR and 1.04 ± 1.37 (range 0–4) logMAR, respectively (*P*=0.20). Vision improved in five eyes, did not change in three eyes, and worsened in one eye. Regarding final VA in the 10 eyes for which final VA data were available, 7 and 4 had final VAs of 20/200 or better and 20/40 or better, respectively.

Cultures of vitreous samples or extracted IOFBs were positive in only two eyes (one for *Staphylococcus epidermidis* and one for *Bacillus cereus*). Retinal tears caused by IOFB impact occurred in four eyes and retinal detachment during IOFB removal in another two eyes. Postoperative complications included a transient increase in intraocular pressure in three eyes and recurrence of retinal detachment and secondary glaucoma in one eye. None of the patients experienced sympathetic ophthalmia during the follow-up period.

The predictors of the final VA are summarized in Table 5. Children with intraoperative RRD had a worse visual recovery than did children with no retinal detachment (Fisher's exact test, *P*=0.0083). Factors such as presence of vitritis, culture results, or IOFB location were not predictors of the final VA.

## 4. Discussion

IOFBs are seen most often in adults and most frequently occur at work sites [3]. However, IOFBs in children are not rare, especially in China. No epidemiological study on the incidence of pediatric IOFBs has been conducted in China. Our hospital, the Eye, Ear, Nose and Throat Hospital of Fudan University, is the largest tertiary referral eye center in East China, and almost all IOFB cases in this region are treated here. Here, we report retrospective interventional follow-up results from 11 children with posterior IOFBs. To our knowledge, this is the first retrospective case series of IOFBs in patients aged < 16 years.

We found that IOFB injuries in children occurred most commonly in the playground, where six children were injured by IOFBs composed of iron, wood, plastic, or stone. One child developed panophthalmitis 1 day after he lit a firework buried in a pile of stones in the playground, an injury that could have been prevented by parental supervision or by wearing protective glasses. To our surprise, our study revealed school as the next most common site for IOFB injury. Four eyes of four children were penetrated by sharp pencils, and the graphite lead remained in the eye after the pencil was removed, which is rarely seen in adults with IOFB. In fact, ocular injuries caused by pencils are not rare in children and include penetration of the eye wall, traumatic cataract, endophthalmitis, and pencil lead retained in the anterior chamber [9] or posterior segment. Pencil fragments left in the eye after orbital or orbitocranial penetrating injuries can even be life and vision threatening [10]. Insufficient awareness of such injuries at school may be the cause of these preventable accidents.

IOFBs were composed of plastic, wood, or pencil lead in six eyes in our study. This supports the suggestion that 50% of IOFBs are not detectable on plain radiographs and is in agreement with the recommendations of other reports that thin-slice CT of the orbits might be preferable [11, 12].

The IOFB was pencil lead in four of the present patients, and was located in the ciliary body in one eye, retina in one eye, and vitreous in one eye. It is debatable whether an IOFB of graphite pencil lead should be removed. Its main component, graphite, can remain inert in the eye for long periods [13]. Indeed, there were no signs of an inflammatory reaction in three cases in our study, even in one case with pencil lead that had remained in the ciliary body for 50 days. However, pencil lead is composed of a mixture of graphite, wax, clay, and animal fat, and potential toxicity due to the other components may lead to progressive damage to ocular structures [14, 15]. Therefore, we removed all pencil lead IOFBs from the eyes in our study, and three of these eyes had a final VA better than 20/30; prognosis was poor in only one eye, as a result of concomitant endophthalmitis and RRD.

During the follow-up period, the VA improved in 5 (of 9) children, and 7 (of 9) children had a final VA better than 20/100. In our study, the OTS sensitivity and specificity to predict visual outcomes was high, which may provide prognostic information in children with open globe injuries, consistent with other studies [16, 17].

The presence of RRD during surgery was a predictor of VA. RRD can be induced during vitrectomy, especially in the presence of severe vitritis and retinitis [3]. Surgical manipulation during extraction of the IOFBs can also lead to dialysis of the ora serrata and thus RRD. In addition, artificial posterior vitreous detachment was difficult in these pediatric patients, as the vitreous gel adheres very tightly to the optic disc, macula, and retinal vasculatures, which could explain the recurrent RRD in one of our patients. Therefore, the surgeon's experience and cautious maneuvers may influence the final prognosis of pediatric patients with IOFBs.

The limitations of this study included the relatively small sample size and lack of a control group, preventing the performance of some statistical analyses and obtaining statistically significant differences. Thus, more studies with larger sample sizes are warranted.

In conclusion, the clinical features of pediatric posterior segment IOFBs were unique, including the IOFB composition and the setting where the injury occurred. The visual prognosis was favorable in this case series, and a poor visual outcome was associated with retinal detachment during removal of IOFB.

## Figures and Tables

**Table 1:** Demographic characteristics of subjects with posterior segment IOFBs.

Subject	Sex	Age (years)	Eye	Interval between accident and surgery (days)	Initial VA	Nature of IOFB	IOFB size (mm)	Location of IOFB	Distance of IOFB to limbus (mm)
1	F	2	OS	2	NA	Pencil tip	5∗1∗1	Ciliary body	5
2	M	10	OD	0.5	20/200	Wood from playground	2∗2∗1.5	Vitreous	14
3	F	5	OD	1	HM	Pencil tip	2∗2∗1	Retina	16
4	M	9	OD	5	20/63	Iron from playground	4∗2∗2	Retina	13
5	M	6	OD	50	20/20	Pencil tip	3∗2∗1	Ciliary body	6
6	M	6	OS	1	NLP	Stone in mud, from exploding fireworks	6∗6∗4	Vitreous	15
7	M	12	OD	0.5	20/160	Iron from playground	5∗3∗3	Retina	16
8	F	6	OD	1	20/200	Pencil tip	2∗2∗1	Vitreous	16
9	M	8	OS	2	LP	Plastic pieces of fireworks	3∗2∗1, 6∗5∗1	Anterior chamber, vitreous	16
10	F	8	OS	5	HM	Iron from hairpin	4∗2∗2	Vitreous	13
11	M	3	OD	6	NA	Iron from playground	3∗1∗1	Vitreous	5

IOFB: intraocular foreign body; VA: visual acuity; OS: left eye; OD: right eye; NA: not available; HM: hand motion; NLP: no light perception; LP: light perception.

**Table 2:** Ocular injuries and OTS in subjects with posterior segment IOFBs.

Subject	Wound entrance	Length of wound (mm)	Anterior chamber and iris	Cataract	Vitreous opacity	Retina	OTS
1	Cornea	1	—	Y	Endophthalmitis	RRD^a^	NA
2	Cornea	3	—	Y	Endophthalmitis	—	3
3	Cornea	5	Iris incarceration	Y	Vitreous hemorrhage	Retinal hole	3
4	Cornea	2	Iris penetration	Y	—	Retinal hole	4
5	Sclera	NA	—	—	—	—	5
6	Cornea	10	Hypopyon, iris dialysis	Y	Panophthalmitis	—	1
7	Sclera	3	—	Y	Vitreous hemorrhage	Retinal hole	4
8	Cornea	5.5	Iris incarceration	Y	—	Retinal hole	4
9	Cornea/sclera	7	Iris incarceration	Y	Endophthalmitis	—	1
10	Cornea	2	Hypopyon	Y	Endophthalmitis	RRD^†^	1
11	Cornea	3	—	Y	—	—	NA

IOFB: intraocular foreign body; OTS: ocular trauma score; RRD: rhegmatogenous retinal detachment; NA: not available. ^†^Injury during IOFB removal.

**Table 3:** Comparison of the ocular trauma score (OTS) predictions with the actual visual outcomes.

		Actual visual outcomes	
		Vision survival	No vision
OTS predictions	Vision survival (LP or better)	6	0
	No vision (NLP or enucleation)	2	1
		Minimal to severe vision loss	Profound vision loss
	Minimal to severe vision loss (20/20∼3/60)	6	0
	Profound vision loss (worse than 3/60)	1	2

OTS: ocular trauma score; LP: light perception; NLP: no light perception.

**Table 4:** Surgeries, complications, and follow-up of subjects with posterior segment IOFBs.

Subject	Primary surgery	IOFB extraction route	Additional surgery	Complications	Final VA	Follow-up (months)
1	LC, PPV, SO	Planar	Retinotomy, SO (3 times)	Glaucoma, RRD	LP	42
2	LC, PPV	Planar	IOL implantation	—	20/25	35
3	LC, PPV, C_3_F_8,_	Planar	IOL implantation	—	20/32	26
4	LC, PPV, C_3_F_8,_	Planar	IOL implantation	IOP increase	20/100	22
5	Anterior PPV	Planar	—	—	20/20	22
6	Evisceration	Evisceration	Hydroxyapatite implantation	—	NLP	22
7	LC, PPV, C_3_F_8,_	Planar	RGP	IOP increase	20/100	20
8	LC, PPV	Planar	IOL implantation	—	20/32	13
9	LC, PPV	Limbal	RGP	—	20/40	9
10	LC, PPV	Limbal	SO removal	IOP increase	HM	8
11	Anterior PPV	Planar	—	—	NA	3

LC: lensectomy; PPV: pars planar vitrectomy; SO: silicone oil tamponade; RRD: rhegmatogenous retinal detachment; VA: visual acuity; LP: light perception; NLP: no light perception; HM: hand motion; IOL: intraocular lens; IOP: intraocular pressure; C_3_F_8_: perfluoropropane; RGP: rigid gas-permeable lens; NA: not available.

**Table 5:** Predictors of final visual outcomes in subjects with posterior segment IOFBs.

Variable	VA ≥ 20/200	VA < 20/200	*P* value (Fisher's exact test)
*Presence of vitritis*			
Endophthalmitis or panophthalmitis	2	3	0.1667
None	5	0	—

*RRD*			
Presence	0	3	0.0083
Absence	7	0	—

*Vitreous or IOFB culture*			
Positive	1	1	1.000
Negative	6	2	—

*Location of IOFB*			
Vitreous	3	2	1.000
Retina or ciliary body	4	1	—

VA: visual acuity; RRD: rhegmatogenous retinal detachment; IOFB: intraocular foreign body.

## Data Availability

The data used to support the findings of this study are available from the corresponding author upon request.
